# Reducing PDK1/Akt Activity: An Effective Therapeutic Target in the Treatment of Alzheimer’s Disease

**DOI:** 10.3390/cells11111735

**Published:** 2022-05-24

**Authors:** Shaobin Yang, Yaqin Du, Xiaoqian Zhao, Chendong Wu, Peng Yu

**Affiliations:** College of Life Sciences, Northwest Normal University, Lanzhou 730070, China; 15682820645@163.com (Y.D.); zxq990726@163.com (X.Z.); chenryoy@163.com (C.W.); pengyu123@nwnu.edu.cn (P.Y.)

**Keywords:** Alzheimer’s disease, amyloid-β, Tau, 3-phosphoinositide dependent kinase 1, protein kinase B, phosphoinositide 3-kinase, TNF-α converting enzyme

## Abstract

Alzheimer’s disease (AD) is a common age-related neurodegenerative disease that leads to memory loss and cognitive function damage due to intracerebral neurofibrillary tangles (NFTs) and amyloid-β (Aβ) protein deposition. The phosphoinositide-dependent protein kinase (PDK1)/protein kinase B (Akt) signaling pathway plays a significant role in neuronal differentiation, synaptic plasticity, neuronal survival, and neurotransmission via the axon–dendrite axis. The phosphorylation of PDK1 and Akt rises in the brain, resulting in phosphorylation of the TNF-α-converting enzyme (TACE) at its cytoplasmic tail (the C-terminal end), changing its internalization as well as its trafficking. The current review aimed to explain the mechanisms of the PDK1/Akt/TACE signaling axis that exerts its modulatory effect on AD physiopathology. We provide an overview of the neuropathological features, genetics, Aβ aggregation, Tau protein hyperphosphorylation, neuroinflammation, and aging in the AD brain. Additionally, we summarized the phosphoinositide 3-kinase (PI3K)/PDK1/Akt pathway-related features and its molecular mechanism that is dependent on TACE in the pathogenesis of AD. This study reviewed the relationship between the PDK1/Akt signaling pathway and AD, and discussed the role of PDK1/Akt in resisting neuronal toxicity by suppressing TACE expression in the cell membrane. This work also provides a perspective for developing new therapeutics targeting PDK1/Akt and TACE for the treatment of AD.

## 1. Introduction

Alzheimer’s disease (AD), a progressive neurodegenerative disorder, currently comprises more than 46.8 million cases worldwide [1,2]. It has two major features, intracerebral neurofibrillary tangles (NFTs) and amyloid-β (Aβ)-related plaque formation, along with a gradual decline in brain functions, leading to memory loss [3]. AD usually affects people over the age of 65 years, and this number is expected to exceed 130 million by 2050 [4,5]. So far, there is no effective therapy to abate its progression. Only five drugs are approved by the US Food and Drug Administration (FDA) (Rivastigmine, Tacrine, Memantine, Galantamine, and Donepezil) to be used for mitigating cognitive impairment in AD patients [6]. However, these treatment strategies fail to prevent the progression of this disease and just delay the onset of the symptoms [7]. Therefore, it is essential to develop drugs that could effectively prevent AD by targeting various molecular pathways associated with this disease.

In neurons, 3-phosphoinositide dependent kinase 1 (PDK1) is responsible for cell survival. PDK1 phosphorylates members of the protein kinase C (PKC) family, serum/glucocorticoid regulated kinase (SGK), protein kinase B (PKB/Akt), p70 ribosomal protein S6 kinase (p70S6K), and p90 ribosomal protein S6 kinase (p90RSK) [8,9]. These signaling pathways regulate proteostasis, the synaptic plasticity of neurons, neuronal polarity, neurotransmission, responses to stresses, and metabolic modulations [10,11,12,13,14]. An increasing amount of evidence has hinted at dysregulated PDK1 expression in AD and altered activation of the PDK1/Akt axis [15,16,17]. Aβ oligomers contribute to hyperactivation of the Akt, and its downstream target proline-rich Akt substrate of 40 kilodaltons (PRAS40) leads to dysregulation of insulin signaling and insulin-like growth factor-1 (IGF-1) resistance in brain cells [18,19]. Akt also regulates the phosphorylation and inactivation of glycogen synthase kinase-3β (GSK-3β), a key step that results in insulin resistance and hyperphosphorylation of Tau protein, leading further to the impairment of brain functions [20,21]. The PDK1/Akt signaling pathway is required for Aβ production and Tau phosphorylation, so studies on the signaling system are necessary for developing an effective therapy for AD.

High PDK1 activity and phosphorylation were found in an autopsied AD brain and mouse brain extracts. These correlate with Src-dependent signaling, which leads to phosphorylation of the TNFα-converting enzyme (TACE) at the threonine (Thr) site and its depletion from the plasma membrane [15]. In 2018, Yang et al. reported that cortical and hippocampal PDK1 phosphorylation at Ser241 in AD mice rises with age, which subsequently increases the phosphorylation of its substrate, Akt, at Thr308 [16]. A mutation in the PDK1 PH-domain affects the phosphorylation of TACE (induced by Aβ), which improves the process of TACE transferring from the cytoplasm to the plasma membrane [16]. This review discusses the possible mechanisms by which the PDK1/Akt/TACE signaling system modulates the physiology and pathology of AD. We begin with an overview of the neuropathological features of AD, then continue with the molecular mechanisms of the regulation of the phosphoinositide 3-kinase (PI3K)/PDK1/Akt pathway, and finally discuss the role of TACE in the pathogenesis of the disease. We also discuss scientific perspectives for the development of new therapeutics targeting PDK1/Akt and TACE dysregulation for effective AD treatment.

## 2. Pathological Features of Alzheimer’s Disease

**Figure 1:**
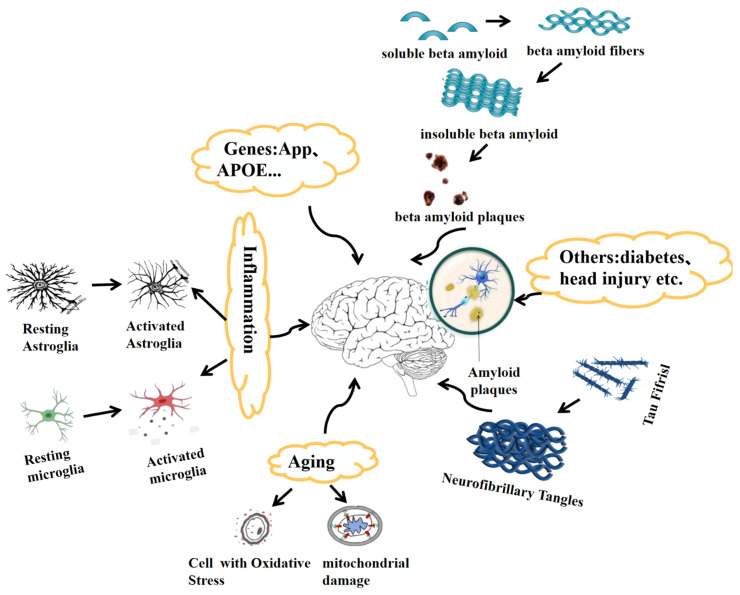
Multifactorial basis of the pathogenesis of Alzheimer’s disease. Hypotheses regarding genetics (APP/Aβ, apoE4), aging, Aβ, Tau, and inflammation have been promoted to explain this multifactorial disorder. Aβ production increases with APP gene mutations, leading to impairment of neuronal activities. Tau forms neurofibrillary tangles and it impairs synaptic functions. Activated microglia and astroglia are important inflammatory factors in AD. Aging increases oxidative stress and mitochondrial dysfunction, which, in turn, improve Aβ production, Tau hyperphosphorylation, and the generation of inflammation. Comorbidities, such as head injury, diabetes mellitus, and other neurodegeneration diseases, could cause AD.

The neuropathological characteristics of senile plaques were initially identified by Dr. Alois Alzheimer as extracellular deposits over 100 years ago. AD is characterized by these plaques, which are an aggregation of misfolded Aβ proteins, and intraneuronal neurofibrillary tangles caused by the accumulation of hyperphosphorylated Tau proteins. Several hypotheses indicate that factors such as genetics, aging, dysregulation of Aβ and Tau, and inflammation play roles in the development of this multifactorial disorder (Figure 1).

### 2.1. Genetics of Alzheimer’s Disease

**Figure 2:**
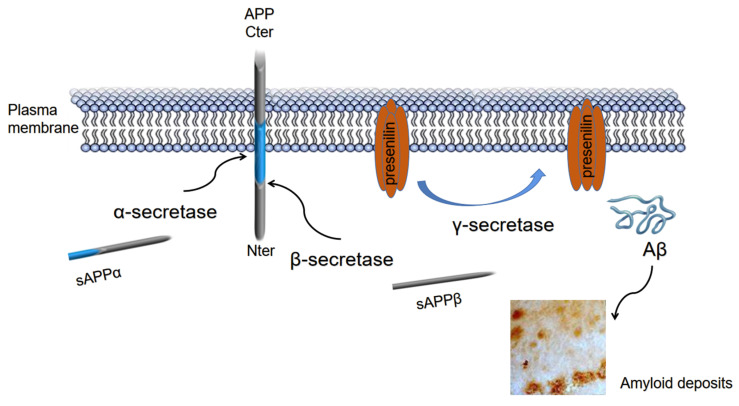
APP’s structure and Aβ processing by α-, β-, and γ-secretases.

A small percentage of AD cases are caused by inherited gene mutations; three such known genes encode the amyloid precursor protein (APP), apolipoprotein E (APOE), and presenilin-1/2 (PSEN1/2) [22,23,24]. The human APP gene has three major isoforms: APP695, APP770, and APP751. APP695 is found on chromosome 21 (21q21.3) and is primarily expressed in the neurons, whereas APP770 and APP751 are found in many tissue samples and in the extracellular region. APP also has one Kunitz protease inhibitor (KPI) domain consisting of 56 amino acids (AAs) [25,26,27,28]. The production of APP in the extracellular region depends on secretase enzymes located on the plasma membrane (PM). The processing of APP occurs in two ways (amyloidogenic and non-amyloidogenic), and it is cleaved by membrane-bound aspartyl proteinases: α-, β-, and γ-secretases. The APP is normally cleaved by α-secretase at the Aβ zone, which yields the sAPPα ectodomain portion and the C-terminal fragment (CTFα) [29,30]. However, sequential cleavage by β-secretases (mediated by BACE1) leads to the release of a soluble fragment (sAPPβ); it then produces a C-terminal peptide that is inserted into the CTFβ membrane [31]. Subsequently, γ-secretase is recruited to cleave CTFβ, resulting in the production of extracellular Aβ peptides (Figure 2). The balance between the elimination and generation of Aβ peptides is known as the amyloidogenic pathway [32,33]. Excessive Aβ production induces synaptic dysfunction, NFT generation, and the eventual death of neurons within AD-related brain regions [7,34,35]. Compared with Aβ40 (a length of 40 residues), Aβ42 is highly hydrophobic and easily becomes pathogenic [36,37,38].

It has been postulated that PSEN1 (PS1) or PSEN2 (PS2) gene mutations are essential components in the occurrence of AD, as they increase the total expression of Aβ and modify the Aβ42:Aβ40 ratio [39,40]. PS genes have two conserved aspartate residues and a cleavage site for the γ-secretase-mediated APP, and produce Aβ in most familiar AD (FAD) patients [41,42]. PS genes are conserved in *Drosophila melanogaster*, *Caenorhabditis elegans*, and lower chordates [43]. In humans and mice, the PS1 mRNA is ubiquitous but it is maximally expressed in the neurons of the hippocampal region of the brain [44,45].

The APOE gene also affects the risk of developing late-onset AD, resulting in synaptic loss and dysregulation of the oxidative stress response and lipid metabolism [46,47,48]. The APOE gene, located on q13.32, can encode a protein of 299 amino acids in human beings [49], and it has an important effect on cholesterol homeostasis [50]. It exhibits several polymorphic forms in humans: the ε2, ε3, and ε4 alleles [49,50]. APOE4 plays a critical role in AD processing, but the ε2 allele provides a protective effect [51,52]. Oligodendrocytes, astrocytes, and neurons secrete APOE into the cerebrospinal fluid (CSF) [53]. The mRNA expression levels of APOE within autopsied AD brains were high compared with those of healthy individuals, but the protein expression levels were inconsistent with this, being higher, lower, or unchanged [53,54,55,56]. APOE affects the lipid metabolism by controlling blood cholesterol levels, and impacts Aβ aggregation, deposition, and elimination [57,58]. It remains unclear how Aβ regulates APOE in AD pathogenesis, and there are no drugs to delay the onset of AD by targeting the apoE4 pathways [59]. This multifactorial disease possibly requires multi-targeted therapy to control AD-related deterioration of brain function.

### 2.2. Tau in Alzheimer’s Disease

The accumulation of phosphorylated Tau is a hallmark of AD. Tau is a critical part of neuronal microtubular networks [60,61,62,63]. The impairment of the axonal transport system caused by abnormal Tau phosphorylation and aggregation disrupts Aβ autophagy [64,65]. Tau is coded by the microtubule-associated tau (MAPT) gene, and it possesses two major domains [66]. The normal microtubule network is composed of Tau, which lays the foundation for axonal transport. This is crucial for regulation of the communication process between the axonal compartments and the somatodendritic regions [66,67]. However, the aggregation of Tau impairs axonal transport, which facilitates Aβ deposition [68,69,70]. p-Tau and Tau oligomer levels rise at the synapses but, surprisingly, no Tau gene mutation has been reported among AD patients [71,72]. The post-translational modification of Tau protein positively correlates with AD development, especially the hyperphosphorylation of Tau [60,61,62,63,64,65,66,67,68,69,70,71,72,73,74]. Most studies have found that Tau accumulation leads to synaptic damage, mitochondrial dysfunction, and neuroinflammation [75,76,77]. Tau pathology also correlates with the status of AD disease. [78,79]. More research on Tau and Aβ pathology would help to develop effective therapies (Figure 1).

### 2.3. Aging as a Factor in Alzheimer’s Disease

Apart from genetic factors, aging is also a risk factor for the development of AD. Some studies have found that aging leads to an increase in Aβ accumulation, cognitive decline, and mitochondrial dysfunction in the brain [80,81,82] (Figure 1). The mitochondrial respiratory chain function and its efficiency are reduced, and the mitochondrial fission–fusion balance, its intracellular movement, and the size of the mitochondria are affected in AD [83,84]. Reactive oxygen species (ROS) and their derivatives not only take part in the onset of AD, as both Aβ plaques and Tau hyperphosphorylation induce their production, but also oxidize polyunsaturated neuronal lipid products [85,86,87]. Clinical and animal studies have shown that the bioenergetic metabolism in the brain is age-dependent; impairment of redox homeostasis, brain hypometabolism, and heightened oxidative stress (ROS) could be observed prior to the occurrence of NFT and Aβ plaque formation [88,89]. However, aging is a complex process and is one of the multiple contributing factors to AD (Figure 1). A deeper understanding of mitochondrial biology in aging and AD could lead to the development of novel therapeutics for treating AD.

### 2.4. Neuroinflammation in Alzheimer’s Disease

Innate immune activation, including the neuroinflammatory response, possibly affects the occurrence of AD and its pathogenicity [90,91,92]. For example, immune receptors such as the cluster of differentiation 33 (CD33), clusterin (CLU), complement receptor 1 (CR1), and the triggering receptor expressed on myeloid cells 2 (TREM2) are related to AD progression, as reported by genome-wide association studies (GWAS) [93]. In AD patients, the abnormal deposition of Aβ leads to activation of the astrocytes and microglia; it results in neuronal damage, as these cells release inflammatory cytokines [94,95,96]. Microglia are neuroprotective under normal physiological conditions, as they release factors that are involved in improving neuronal circuits as well as tissue maintenance, thus facilitating synapse remodeling [97,98]. Neuronal death or protein aggregation could act as pathological triggers that activate microglial cells during the presymptomatic stage of AD. Activated microglia release proinflammatory factors such as tumor necrosis factor-α (TNF-α), interleukin-1α (IL-1α), and interleukin-1β (IL-1β) [99,100,101]. Thereafter, astrocytes, with excessive reactivity, are recruited around senile plaques, which can be observed in brain tissues of AD patients post-mortem or in animal models of AD [102,103]. Proinflammatory and neuroinflammatory cytokines increase Tau phosphorylation and decrease synaptophysin levels [104,105]. However, anti-inflammatory drugs and compounds to treat or prevent AD have failed due to the complexity of this disease [106]. Therefore, a combination of therapeutics will have a high potential for treating or inhibiting AD’s progression (Figure 1).

## 3. Structure and Function of the PI3K/PDK1/Akt Signaling Pathway

### 3.1. Structure and Function of PI3K

The phosphoinositide 3-kinase (PI3K) family has an important role in cell growth, migration, survival, and cytoskeletal changes; in mammals, this family is divided into three classes (1A and 1B, 2, and 3) [107]. Class 1A possesses one catalytic and one regulatory subunit; the former includes p110α/β/δ isoforms, while the latter includes the p85α, p85β, and p55γ isoforms [107]. Class 1B consists of two regulatory subunits, p101 and p87, but only one catalytic subunit, p110γ [108]. The Class 1 (A and B) PI3Ks are recruited to the cell membrane in response to the extracellular signals transmitted by the growth factor receptors, which then phosphorylate the phospholipid, phosphatidylinositol-4,5-bisphosphate (PtdIns (4,5) P2), at the C-terminal end to produce phosphatidylinositol-3,4,5-trisphosphate (PtdIns (3,4,5) P3) [109]. Moreover, there are three isoforms in Class 2, including PI3K2α/2β/2γ, which has a C-terminal extension formed by the C2 and PX domains, which is responsible for its association with PtdIns (4,5) P2-containing plasma membranes [110]. There is just a single isoform in Class 3, hVps34 [111].

PI3Ks of Class 1 can promote protein–membrane transport and activate the protein to be a second messenger molecule [112,113]; the serine/threonine Akt is one such molecule. In contrast, pentaerythritol tetranitrate (PETN), a lipid phosphatase, antagonizes PI3K activity [114,115]. Akt has a pleckstrin homology (PH) domain that promotes the translocation of Akt to the plasma membrane by binding to PtdIns (3,4,5) P3. PDK can phosphorylate Akt at the threonine position (Thr308) inside an activation loop [116]. However, the second phosphorylation of Akt at the Ser473 position is catalyzed by Ser/Thr kinase and the mammalian target of rapamycin (mTORC2), which is itself activated directly by PtdIns (3,4,5) P3 [117]. To regulate the activities within the cell, Akt phosphorylates numerous downstream substrates such as the cAMP-dependent protein kinase A/cGMP-dependent protein kinase G/protein kinase C (AGC) family of serine/threonine kinases, p70 ribosomal S6K kinase (S6K), RSK, SGK, and PKC [118,119]. The AGC group members require phosphorylation at two sites, one of which is inside a kinase domain, which is the turn motif/zipper phosphorylation site in the activation loop; to be completely activated, it requires phosphorylation at a site in another catalytic domain adjacent to the hydrophobic motif (HM) [8].

### 3.2. Structure and Function of PDK1

**Figure 3:**
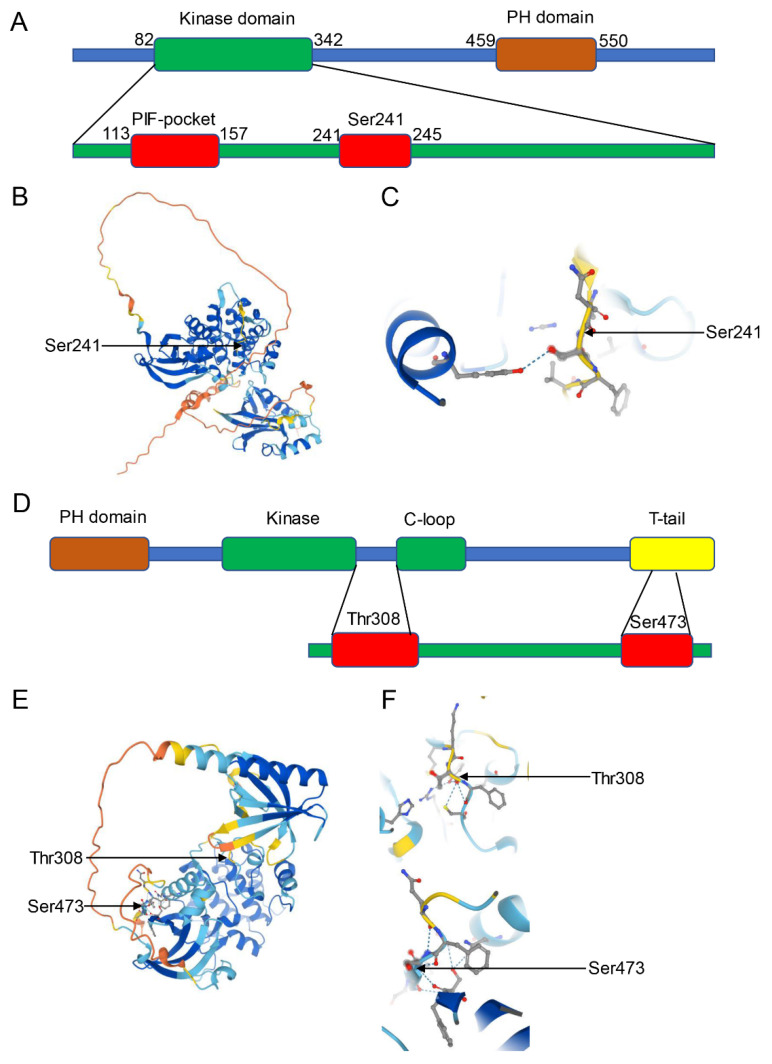
(**A**) The general structure of the peptide chain of PDK1. (**B**) Structural features of the catalytic core of PDK1. Ser241 represents the phosphorylation site inside the activation loop. (**C**) Structural features of the Ser241 of PDK1. (**D**) The general structure of the peptide chain of Akt. (**E**) Structural features of the catalytic core of Akt. Ser473 in the regulating domain and Thr308 in the catalytic domain of the activation loop represent the phosphorylation site. (**F**) Structural features of the Ser473 and Thr308 of Akt.

PDK1 is the kinase that is responsible for the PtdIns (3,4,5) P3-dependent Akt phosphorylation at the Thr308 position [120]. PDK1 is an upstream activator that can specifically phosphorylate the Ser or Thr residue in the T-loop of 23 members of the AGC kinase family, including p70S6K, SGK, p90RSK, and PKC [121]. PDK1 consists of 556 amino acids and has two domains, namely one PH domain at the C-terminal and one catalytic domain at the N-terminal; the structural organization of these domains is completely conserved in the animal kingdom [122]. PDK1 possesses two important regulatory elements that are required for kinase activity [123]. Ser241 represents the phosphorylation site inside the activation loop, and PDK1 can catalyze Ser241 in trans for its kinase activity [124]. The PIF pocket is localized in the domain at the N-terminal―an important domain consisting of a conserved phosphorylated motif (Figure 3A).

PDK1 itself is constitutively catalyzed inside its activation loop in various positions, including Ser241 [125]. It occurs constitutively, suggesting that trans-phosphorylation and homo-dimerization possibly fine-tune PDK1’s activity [126] (Figure 3B,C). Several mechanisms are involved in PDK1-mediated signal modulation; the PDK1-mediated Akt phosphorylation at Thr308 in the PH domain is an important event and leads to the activation of Akt [127]. However, to be completely active, the phosphorylation of Ser473 at the HM needs to be mediated by mTORC2 [128]. A different mechanism activates SGK and S6K kinases compared with Akt activation; these kinases lack the PH domain but possess the Ser/Thr residue within the HM, along with the PDK1 proteolysis-inducing factor (PIF)-binding pocket. The PDK1 catalytic domain exists in a typical groove inside the PIF pocket (a tiny lobe), and this provides a site for substrate docking, improving the anchoring and phosphorylation of PDK1 on the corresponding substrates [129]. These HMs contain conserved Phe-Xaa-Xaa-Phe/Tyr-Ser/Thr-Phe/Tyr sequence, whereas Ser/Thr plays a critical role in maximally activating the enzymes [130]. Moreover, AGC kinase phosphorylation within the HM can promote PDK1’s interaction with the corresponding substrates [131]. Therefore, PI3K enhances the activation of SGK and S6K by regulating the phosphorylation of the related enzymes within the HM (Figure 3).

### 3.3. Structure and Function of Akt

As a serine/threonine protein kinase, Akt represents the downstream molecule in the PI3K pathway, which critically regulates different cell activities [132,133]. Mammals have three distinct Akt isoforms encoded by distinct genes, namely Akt1, Akt2, and Akt3, all of which are tightly associated [134]. Akt isoforms share 85% amino acids as well as the structure of three domains, a central catalytic kinase domain, an N-terminal PH domain, and a regulating domain at the C-terminal that exists within the HM [116,135]. These domains are highly conserved in animals, including worms, flies, mice, and humans [136]. When PI3K becomes activated, PIP3 binds to its PH domain to fully activate Akt (Figure 3D). Thus, the Thr308, Thr309, or Thr305, in the catalytic domain of the activation loop facilitate the recruitment of PDK1 to the membrane, and phosphorylation at the Ser473, Ser474, or Ser472 site of the regulating domain within the HM is needed [8] (Figure 3E,F).

The phosphorylation of Thr308 is required to achieve a conformation that renders Akt fully active. The phosphorylation of Ser473 is needed for inter-HM intramolecular interactions [124]. The serine/threonine residues of other Akt molecules could be phosphorylated by activated Akt within the sequence Arg-X-Arg-X-X-Ser/Thr-Hyd, with X and Hyd representing any amino acid and bulk hydrophobic residue, respectively [137]. On the other hand, glycogen synthase kinase-3 (GSK-3) is a downstream member of the PI3K–Akt axis, and it has two competitive inhibitory sites: GSK-3α at Ser21 and GSK-3β at Ser9, which are phosphorylated by Akt [138,139]. GSK3 plays an important physiological role in glial and neuronal cell functions; it can regulate various neuronal processes such as protein production and degradation [140,141]. The activation of FOX family members is suppressed by the PI3K/PDK1/Akt signaling pathway [142]. Overexpressed and overactive (forkhead box group O transcription factor) FoxO leads to cell apoptosis [143]. The Akt signaling axis mediates numerous physiological cell events such as cell growth, survival, proliferation, and metabolism, and its dysregulation results in tumors or neurodegenerative disorders.

## 4. The PI3K/PDK1/Akt Pathway in Normal and AD Brains

### 4.1. The PI3K/PDK1/Akt Pathway in Normal Brains 

**Figure 4:**
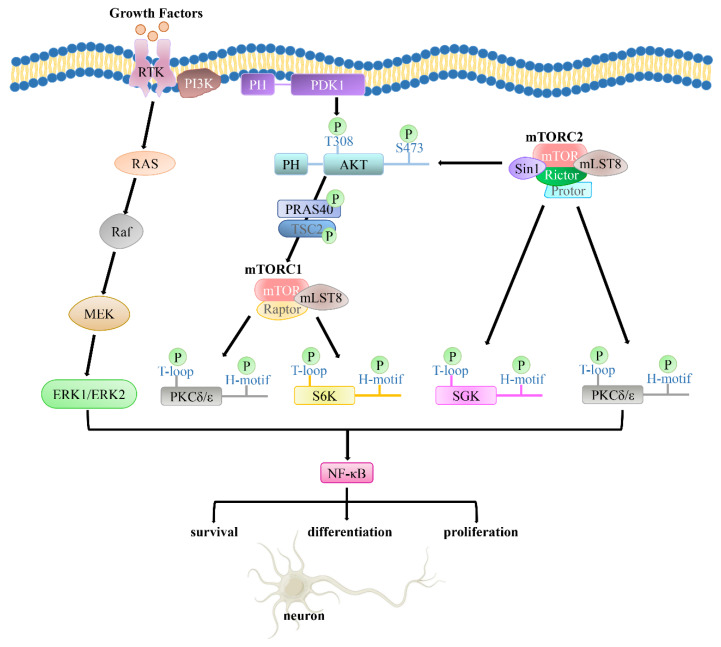
The PI3K/PDK1/Akt pathway plays a crucial role in cell functions, such as neuronal survival, differentiation, and proliferation, which contribute to neuronal myelination, migration, and the establishment of neuronal polarity.

Insulin and IGF-1 regulate neuronal survival, differentiation, proliferation, and motility, facilitating neuronal migration and myelination as well as the development of neuronal polarity via phosphorylation and dephosphorylation of the adaptor proteins, depending on the PI3K/PDK1/Akt signaling pathway [10,11] (Figure 4). The PI3K/Akt pathway plays important roles in the differentiation, polarity development, and survival of neurons, along with synaptic plasticity, neurotransmission through the axon–dendrite axis, metabolic control, and stress responses within adult brains [144] (Figure 4). Brain-derived neurotrophic factor (BDNF) enhances the length and complexity of dendrites by activating the PI3K/Akt signaling pathway, which plays an important role in microtubule transport and the maintenance of synaptic plasticity [145,146]. The PI3K signaling pathway also mediates γ-aminobutyric acid (GABA) receptor trafficking and phosphorylation [147]. This signaling axis is responsible for long-term potentiation (LTP), essential protein biosynthesis, signal transmission between neurons, and memory consolidation [148].

### 4.2. The PI3K/PDK1/Akt Pathway in AD Brains

Akt is increasingly activated in the hippocampal and cortical neurons of AD patients, and its subcellular localization is changed with concomitant inactivation of PTEN [149,150,151]. This hyperactivation of Akt builds resistance against IGF-1 and insulin in AD neurons utilizing the c-Jun N-terminal kinase (JNK)–tumor necrosis factor-α (TNFα) pathway [152,153,154]. Incessant activation of the PI3K/Akt pathway suppresses mTOR inhibition and the protective effect of FOXO signaling, thus aggravating the effects of Tau hyperphosphorylation and Aβ deposition, cognition impairment, and synaptic damage [153]. Dysfunction of the PI3K/Akt signaling axis also causes the phosphorylation of GSK-3β [140]. Hyperphosphorylation of Tau accounts for the formation of paired helical filaments in AD, which accumulate in degenerating neurons [154,155]. However, some studies have found that Aβ can inhibit activation of the PI3K/Akt pathway while removing the suppression of GSK-3β phosphorylation [156]. The activation of the PI3K/Akt pathway prevents hyperphosphorylation of Tau by disrupting the insulin signaling axis [157]. Therefore, the PI3K/Akt pathway is an initial effector of Aβ-Tau’s pathophysiological responses; it also controls neuronal development and its functions, and it is implicated in deficient cognitive behavior [158].

Yang et al. found that in APP/PS1 and 3xTg-AD mice, PDK1 phosphorylation at the Ser241 position, together with Akt phosphorylation at the Thr308 position, increases in cortical and hippocampal tissues with aging [16]. PDK1 autophosphorylation at Ser241 is important but fails to induce the complete activation of PDK1 [159]. Additional phosphorylation of the Ser, Tyr, and Thr residues in the kinase domain is required to fully activate the PDK1 [159]. PDK1 autophosphorylates itself at Ser241 with insulin signaling. Besides, PDK1 phosphorylation at the Tyr-9/-373 positions in the presence of heat shock protein 90 (Hsp90) and Src are significant for enhancing the catalytic performance of PDK1 [159]. AD is sometimes referred to as Type III diabetes, as dysregulation of insulin and glucose metabolism are seen in patients [158]. Furthermore, the Akt phosphorylation at the Ser473 position significantly rises within the hippocampus and cortices in 3xTg-AD mice [16]. Another study has also found that Aβ could lead to mTORC1 hyperactivity in the hippocampus of AD mice [18]. Increased phosphorylation of PRAS40 at the Thr246 position leads to Akt hyperactivity in AD [160], and this was also found in the brain of aged mice [16]. After treatment with the growth factor, Akt activity was upregulated in aged fruit flies [161]. In contrast, the use of RNAi downregulates Akt phosphorylation and decreases neuronal death, improving the starvation condition and locomotor activity in aged and Aβ42-induced flies [161]. Taken together, Aβ accumulation enhances PI3K/PDK1/Akt pathway activity and increases the phosphorylation of PRAS40, which results in mTORC1 hyperactivity.

The GSK3β phosphorylation at Ser9 increases in the hippocampus and cortices in old AD mice and in Aβ42 flies [161]. The phosphorylation of FOXO at Ser256 is also improved in AD [16]. However, the hyperphosphorylation of FOXO reverses the Aβ42-induced death of neurons and the impairment of learning abilities in flies [161]. Collectively, these findings indicate that the PDK1/Akt signaling axis is hyperactivated due to AD pathology.

Mitogen-activated protein kinase (MAPK) plays an important role in cell survival and apoptosis, mitogenesis, and differentiation in neurons [162]. In AD, the activation of MAPK is remarkedly associated with Tau hyperphosphorylation, and Aβ leads to an increase in intracellular calcium and mitochondrial stress through activation of MAPK in the neurons [163,164,165]. The inhibition of the MAPK pathway could reduce Aβ-induced neurotoxicity and apoptosis [166,167]. 

The nuclear factor-κB (NF-κB) transcription factor is a key regulator of neuron survival and apoptosis, differentiation, neurite proliferation, and synaptic plasticity [168]. NF-κB upregulates the generation oof Aβ via increasing transcription of the BACE1 gene [169,170,171]. Aβ42 induces neuroinflammation and oxidative stress, which depend on NF-κB in the brain [172]. Therefore, AD could be improved by inhibiting the expression of NF-κB. On the basis of this theory, the crosstalk between the PI3K/PDK1/Akt and MAPK/NF-κB pathways should be explored further, which might be helpful for the prevention and treatment of AD.

## 5. The Therapeutic Possibilities of the Modulation of PDK1/Akt in AD

### 5.1. The Therapeutic Possibility of Mutation of the PDK1 PH-Domain in AD

Due to the significant effect of the PI3K/PDK1/Akt signaling pathway on AD deterioration, the activation or suppression of this signal can be used to treat AD, depending on the stage of the disease. Leptin and lithium are two compounds that can alleviate AD by mediating Akt phosphorylation at the Ser473 position [158,172,173,174,175]. However, there are no reports about any exogenous compounds that affect the phosphorylation of Akt at Thr308 and of PDK1 at Ser241 in AD.

PDK1 K465E mice can be treated by introducing the lysine 465-to-glutamic acid mutation in the PH domain of PDK1, as the K465E mutation markedly reduces the positively-charged ligand-binding interface by damaging the lipid-binding pocket’s conformation [176]. Phosphorylation at the Thr308 position of PDK1^K465E/K465E^ mice was lower in islets or skeletal muscle than in those of control mice after being injected with insulin [177]. Akt phosphorylation decreases within embryonic neurons present in the cortices of PDK1^K465E/K465E^ mice after being stimulated by brain-derived neurotrophic factor (BDNF) activation [178]. According to several studies, PDK1-to-phosphoinositides binding plays a critical role in activating Akt [168]. However, the phosphorylation of Akt at Ser473 remained unaffected by this PDK1 mutation [178]. Aβ might improve the production of BDNF within PDK1^K465E/K465E^ brains and neurons [16]. Importantly, in the older PDK1^K465E/K465E^ mice, the BDNF content within the cortices and hippocampi rose [16]. It is possible that phosphoinositide binding does not mediate PDK1 protein, because PDK1 catalytic activity was intact in the brains of PDK1^K465E/K465E^ mice.

Najafov et al. found that Akt protein can be phosphorylated depending on the PIF pocket or PH domain. The dual pathway may promote the efficient activation of Akt [169]. The PIF pocket in PDK1 is responsible for the phosphorylation of Akt at the Thr308 position without binding PDK1 to PtdIns (3,4,5) P3 [179]. This is seen frequently in brain tissues compared with other tissue types, possibly due to the existence of the BDNF/tropomyosin-related kinase (TrkB) pathway [180]. PDK1 can particularly bind to PIF peptides, which indicates the phosphorylation of Akt at the Thr308 position in the brains of PDK1^K465E/K465E^ mice, thus achieving phosphorylation at Ser373 [16].

Both young and mature PDK1^K465E/K465E^ mice improved cognitive deficits, as tested by a spatial working memory task, indicating that the Akt signaling axis is more effective in the young brain than in adulthood [181]. Specific patterns related to age are elicited in the hippocampus and cortex, suggesting that the quantitative/qualitative variations in neural circuit disruption induce the different phenotypes associated with certain mental diseases [182]. According to the results, adjusting the Akt signaling can induce different physiological reactions as well as their readout in vivo [182]. Finally, this study also found a similar initial fear response and horizontal locomotion in PDK1^K465E/K465E^ mice, both males and females, but only females showed elevated thigmotaxis, a reduced latency to approach the periphery while performing wall rearing, and exhibited faster ethograms than males [181,182]. In summary, the distinct behavior characteristics due to genotype, age, and sex suggest that these factors influence PI3K/Akt pathway for eliciting diverse behaviors.

Since the PI3K/PDK1/Akt signaling pathway plays an important role in the progression of AD, new drugs used to treat the disease are based on the regulation of this signaling axis. It has been found that the suppression or activation of the Pl3k/PDK1/Akt signaling by some compounds in AD depends on the stage and the type of cells in this degenerative disease [158]. Some promising candidates that could be therapeutic targets for the modulation of the PI3K/PDK1/Akt signaling axis are summarized in Table 1. However, most of the compounds, including *Salvia officinalis* and curcumin, are limited in their application because of bioavailability; leptin and lithium exhibit other defects through activating the PI3K/Akt signaling pathway [138,183,184,185]. Rapamycin and AZD8055 were used as treatment strategies but also failed, given the emergence of several side effects [186,187]. Inhibiting PDK1 with BX912 could reduce the pathology of AD in mice, but led to mice dying because of BX912 toxicity [15]. Consequently, it may be a promising strategy to develop more effective and safer compounds with PDK1/Akt targets.

### 5.2. The Therapeutic Possibility of Mutating PDK1’s PH-Domain Dependence with TACE in AD

TACE belongs to the A disintegrin and metalloproteinase (ADAM) family; its secretase is known for the shedding of transmembrane pro-TNFα into soluble forms (sTNFα) [193]. TACE also uses α-secretase for cleavage of the amyloid precursor and TNFR1 in neurons, thereby improving the levels of soluble TNFR1 and Aβ production [193]. The levels of sAPPα and sTNFR1 decrease within the CSF in AD cases due to the deregulation of the TACE α-secretase, which also has a critical effect on prion neuropathogenesis [194]. The PrPSc-induced over-activation of PDK1 decreases TACE-mediated α-secretase expression and favors the β-processing of APP, leading to Aβ40/42 accumulation [195]. Pietri et al. found that PDK1 is the downstream molecule of the Src-mediated pathway and facilitates TACE phosphorylation and its consumption from the membrane in AD patients [15]. Pharmacological inhibition and PDK1 siRNA decrease the levels of Aβ and reverse TACE-mediated sAPPα expression. These processes also enhance TACE-induced α-secretase expression while reducing memory impairment and the pathology of AD in mice [15]. These studies have shown that the dysregulation of the PDK1–TACE–APP signaling pathway accelerates the accumulation of Aβ.

According to Yang et al., the levels of sTNFR1 and TACE-induced α-secretase are markedly higher in PDK1^K465E/K465E^ mouse brains compared with control mice of a similar age [16]. In particular, their expression is significantly elevated in PDK1^K465E/K465E^ neurons after being treated with Aβ [16]. The phosphorylation of TACE at Thr735 is reduced in the brains of PDK1^K465E/K465E^ mice. Therefore, inhibiting the binding of PDK1-PtdIns (3,4,5) P3 in PDK1’s PH-domain reduces TACE’s activity and its phosphorylation [16]. As reported by Chen et al., Akt regulates γ-secretase, which affected APP processing by modulating Notch activity in flies [161]. These mechanisms could synergistically explain that Aβ resistance is dependent on the upregulation of TACE in PDK1^K465E/K465E^ neurons [16]. This main characteristic of mutation of the PDK1 PH-domain is similar to that of Akti-1/2 compounds such as Akt1/Akt2, which show specific expression patterns in brain tissues [196]. This compound is currently being tested as an oral therapeutic agent in clinical trials to treat certain cancers [197]. These studies provide genetic verification that Akt inhibitors can be used for treating AD.

## 6. Conclusions

**Figure 5:**
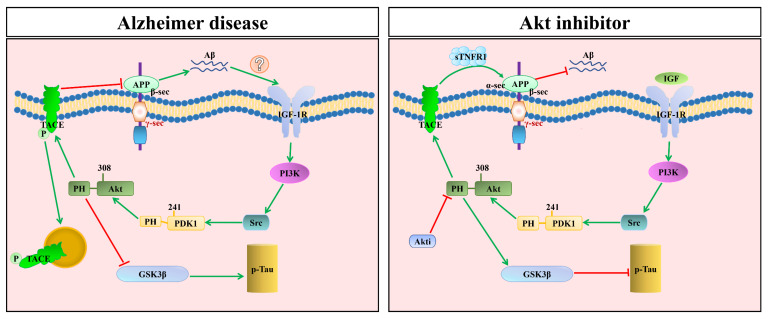
In Alzheimer’s disease, the activity of APP γ-secretase could lead to APP cleavage and generate Aβ, increasing the activation of PI3K and the phosphorylation of PDK1 at Ser241 and Akt at Thr308 and Ser473, then phosphorylating TACE. This process induces the internalization of TACE in the caveolae, thereby reducing the TACE-mediated production of sTNFR1. If Akt inhibitor is used at the Thr308 site, this reduces the phosphorylation of TACE, which preserves all the TACE at the membrane, increases the TACE-mediated production of sTNFR1, and decreases the accumulation of Aβ.

AD is a multifactorial neurodegenerative disorder with high complexity. Typically, APP, PS1, PS2, APOE, Aβ aggregation, Tau hyperphosphorylation, neuroinflammation, and aging are responsible for the onset and development of AD. The PDK1/Akt signaling axis is associated with cell survival. Aging improves the activation of the PDK1/Akt signaling axis and decreases the lifespan, producing low stress resistance, locomotion defects, learning/memory impairment, cellular death, and aging-related pathologies. However, the reduced expression of intracerebral PDK1/Akt reduces the abovementioned pathological phenotypes while mitigating Aβ-mediated injury. The dual roles of PDK1/Akt in the brain suggest the possible existence of a positive feedback loop between AD and the PDK1/Akt pathway. Generally, aging can enhance the expression of Akt for promoting γ-secretase expression, resulting in Aβ generation and APP cleavage. The upregulated Aβ levels can further activate the Akt and PDK1 phosphorylation rate, enhancing γ-secretase expression, which promotes APP processing, but reduces TACE’s α-secretase activity (Figure 5). PDK1/Akt may have separate molecular activities in different cell types. In summary, we reviewed the relationship between the PDK1/Akt signaling pathway and AD and discussed the possibility that reducing the rate of activation of PDK1/Akt can prevent Aβ-mediated neuronal toxicity by suppressing the expression of TACE in the cell membrane. These scientific findings will hopefully lead to the development of novel therapeutics for treating AD.

## Figures and Tables

**Table 1:** List of chemical compounds used for the treatment of AD.

Compounds	Molecular Mechanisms	References
Salvia officinalis	Activates PI3K/Akt	[183]
Curcumin	Activates PI3K/Akt/mTOR	[184]
Leptin	Activates PI3K/Akt	[138,139]
Tripchlorolide	Activates PI3K/Akt/mTOR	[188]
Achyranthes	Activates PI3K/Akt	[189]
Lithium	Activates the PI3K/Akt axis	[185]
Rapamycin	Inhibits mTORC1	[186]
PI103	Inhibits mTORC1	[190]
AZD8055	Inhibits mTORC1 and mTORC2	[187]
INK128	Inhibits mTORC1 and mTORC2	[191]
BX912	Inhibits PDK1	[15]
OSU03012	Inhibits PDK1	[192]

## References

[1] Ijaopo E.O. (2017). Dementia-related agitation: A review of non-pharmacological interventions and analysis of risks and benefits of pharmacotherapy. Transl. Psychiatry.

[2] Dementia Statistics Alzheimer’s Disease International (ADI). https://www.alzint.org/about/dementia-factsfigures/dementia-statistics/.

[3] Murphy M.P., Le Vine H. (2010). Alzheimer’s disease and the amyloid-peptide. J. Alzheimer’s Dis..

[4] McKhann G.M., Knopman D.S., Chertkow H., Hyman B.T., Jack C.R., Kawas C.H., Klunk W.E., Koroshetz W.J., Manly J.J., Mayeux R. (2011). The diagnosis of dementia due to alzheimer’s disease: Recommendations from the national institute on aging- alzheimer’s association workgroups on diagnostic guidelines for alzheimer’s disease. Alzheimer’s Dement..

[5] (2020). Alzheimer’s disease facts and figures. Alzheimer’s Dement..

[6] Auld D.S., Kornecook T.J., Bastianetto S., Quirion R. (2002). Alzheimer’s disease and the basal forebrain cholinergic system: Re-lations to β-amyloid peptides, cognition, and treatment strategies. Prog. Neurobiol..

[7] Farlow M.R., Miller M.L., Pejovic V. (2008). Treatment options in Alzheimer’s disease: Maximizing benefit, managing expectations. Dement. Geriatr. Cogn. Disord..

[8] Bayascas J.R. (2010). PDK1: The Major Transducer of PI 3-Kinase Actions. Tuberculosis.

[9] Alessi D.R., Deak M., Casamayor A., Caudwell F.B., Morrice N., Norman D.G., Gaffney P., Reese C.B., MacDougall C.N., Harbison D. (1997). 3-Phosphoinositide-dependent protein kinase-1 (PDK1): Structural and functional homology with the Drosophila DSTPK61 kinase. Curr. Biol..

[10] Ye L., Wang X., Cai C., Zeng S., Bai J., Guo K., Fang M., Hu J., Liu H., Zhu L. (2019). FGF21 promotes functional recovery after hypoxic-ischemic brain injury in neonatal rats by activating the PI3K/Akt signaling pathway via FGFR1/β-klotho. Exp. Neurol..

[11] Vanhaesebroeck B., Guillermet-Guibert J., Graupera M., Bilanges B. (2010). The emerging mechanisms of iso-form-specific PI3K signalling. Nat. Rev. Mol. Cell Biol..

[12] Wei Y., Han X., Zhao C. (2020). PDK1 regulates the survival of the developing cortical interneurons. Mol. Brain.

[13] Kim H., Lee J., Cho Y. (2021). PDK1 is a negative regulator of axon regeneration. Mol. Brain.

[14] Xu C., Yu L., Hou J., Jackson R.J., Wang H., Huang C., Liu T., Wang Q., Zou X., Morris R.G. (2017). Conditional Deletion of PDK1 in the Forebrain Causes Neuron Loss and Increased Apoptosis during Cortical Development. Front. Cell. Neurosci..

[15] Pietri M., Dakowski C., Hannaoui S., Alleaume-Butaux A., Hernandez-Rapp J., Ragagnin A., Mouil-let-Richard S., Haik S., Bailly Y., Peyrin J.M. (2013). PDK1 decreases TACE-mediated α-secretase activity and promotes disease progression in prion and Alzheimer’s diseases. Nat. Med..

[16] Yang S., Pascual-Guiral S., Ponce R., Gimenez-Llort L., Baltrons M., Arancio O., Palacio J.R., Clos V.M., Yuste V.J., Bayascas J.R. (2018). Reducing the levels of Akt activation by PDK1 knock-in mutation protects neuronal cultures against synthetic amyloid-beta peptides. Front. Aging Neurosic..

[17] Rao C.V., Farooqui M., Madhavaram A., Zhang Y., Asch A.S., Yamada H.Y. (2020). GSK3-ARC/Arg3.1 and GSK3-Wnt signaling axes trigger amyloid-β accumulation and neuroinflammation in middle-aged Shugoshin 1 mice. Aging Cell.

[18] Caccamo A., Maldonado M.A., Majumder S., Medina D.X., Holbein W., Magrí A., Oddo S. (2011). Naturally secreted amyloid-beta increases mammalian target of rapamycin (mTOR) activity via a PRAS40-mediated mechanism. J. Biol. Chem..

[19] Velazquez R., Shaw D.M., Caccamo A., Oddo S. (2016). Pim1 inhibition as a novel therapeutic strategy for Alzheimer’s disease. Mol. Neurodegener..

[20] Lauretti E., Dincer O., Praticò D. (2020). Glycogen synthase kinase-3 signaling in Alzheimer’s disease. Biochim. Biophys. Acta Mol. Cell Res..

[21] Zhang Y., Huang N.Q., Yan F., Jin H., Zhou S.Y., Shi J.S., Jin F. (2018). Diabetes mellitus and Alzheimer’s disease: GSK-3beta as a potential link. Behav. Brian Res..

[22] Campion D., Dumanchin C., Hannequin D., Dubois B., Belliard S., Puel M., Thomas-Anterion C., Michon A., Martin C., Charbonnier F. (1999). Early-Onset Autosomal Dominant Alzheimer Disease: Prevalence, Genetic Heterogeneity, and Mutation Spectrum. Am. J. Hum. Genet..

[23] Bertram L., Lill C.M., Tanzi R.E. (2010). The Genetics of Alzheimer Disease: Back to the Future. Neuron.

[24] Serrano-Pozo A., Das S., Hyman B.T. (2021). APOE and Alzheimer’s disease: Advances in genetics, pathophysiology, and thera-peutic approaches. Lancet Neurol..

[25] Dorszewska J., Prendecki M., Oczkowska A., Dezor M., Kozubski W. (2016). Molecular basis of familial and sporadic Alzheimer’s disease. Curr. Alzheimer Res..

[26] Cohen I., Coban M., Shahar A., Sankaran B., Hockla A., Lacham S., Caulfield T.R., Radisky E.S., Papo N. (2019). Disulfide engineering of human Kunitz-type serine protease inhibitors enhances proteolytic stability and target affinity toward mesotrypsin. J. Biol. Chem..

[27] Wasco W., Gurubhagavatula S., Paradis M.D., Romano D.M., Sisodia S.S., Hyman B.T., Neve R.L., Tanzi R.E. (1993). Isolation and characterization of APLP2 encoding a homologue of the Alzheimer’s associated amyloid beta protein precursor. Nat. Genet..

[28] Coulson E.J., Paliga K., Beyreuther K., Masters C.L. (2000). What the evolution of the amyloid protein precursor supergene family tells us about its function. Neurochem. Int..

[29] Checler F., Afram E., Pardossi-Piquard R., Lauritzen I. (2021). Is γ-secretase a beneficial inactivating enzyme of the toxic APP C-terminal fragment C99?. J. Biol. Chem..

[30] Mattson M.P. (1997). Cellular actions of beta-amyloid precursor protein and its soluble and fibrillogenic derivatives. Physiol. Rev..

[31] Cai H., Wang Y., McCarthy D., Wen H., Borchelt D.R., Price D.L., Wong P.C. (2001). BACE1 is the major beta-secretase for generation of Abeta peptides by neurons. Nat. Neurosci..

[32] Miranda A., Montiel E., Ulrich H., Paz C. (2021). Selective Secretase Targeting for Alzheimer’s Disease Therapy. J. Alzheimer’s Dis..

[33] Hampel H., Vassar R., De Strooper B., Hardy J., Willem M., Singh N., Zhou J., Yan R., Vanmechelen E., De Vos A. (2021). The beta-Secretase BACE1 in Alz-heimer’s Disease. Biol. Psychiatry.

[34] Gonzalez C., Armijo E., Bravo-Alegria J., Becerra-Calixto A., Mays C.E., Soto C. (2018). Modeling amyloid beta and tau pathology in human cerebral organoids. Mol. Psychiatry.

[35] Shankar G.M., Walsh D.M. (2009). Alzheimer’s disease: Synaptic dysfunction and α-beta. Mol. Neurodegener..

[36] Dal Prà I., Chiarini A., Gui L., Chakravarthy B., Pacchiana R., Gardenal E. (2015). Do astrocytes collaborate with neurons in spreading the “infectious” Aβ and tau drivers of Alzheimer’s disease?. Neuroscientist.

[37] Kurz A., Perneczky R. (2011). Novel insights for the treatment of Alzheimer’s disease. Prog. Neuro-Psychopharmacol. Biol. Psychiatry.

[38] Gillardon F., Rist W., Kussmaul L., Vogel J., Berg M., Danzer K., Kraut N., Hengerer B. (2007). Proteomic and functional alterations in brain mitochondria from Tg2576 mice occur before amyloid plaque deposition. Proteomics.

[39] Fedeli C., Filadi R., Rossi A., Mammucari C., Pizzo P. (2019). PSEN2 (presenilin 2) mutants linked to familial Alzheimer disease impair autophagy by altering Ca^2+^ homeostasis. Autophagy.

[40] Fung S., Smith C.L., Prater K.E., Case A., Green K., Osnis L., Winston C., Kinoshita Y., Sopher B., Morrison R.S. (2020). Early-onset familial Alzheimer disease variant PSEN2 N141I heterozygosity is associated with altered microglia Phenotype. J. Alzhemers Dis..

[41] Lanoiselée H.M., Nicolas G., Wallon D., Rovelet-Lecrux A., Lacour M., Rousseau S., Richard A.C., Pasquier F., Rollin-Sillaire A., Martinaud O. (2017). APP, PSEN1, and PSEN2 mutations in early-onset Alzheimer disease: A genetic screening study of familial and sporadic cases. PLoS Med..

[42] Qiu Q., Jia L., Wang Q., Zhao L., Jin H., Li T., Quan M., Xu L., Li B., Li Y. (2020). Identification of a novel PSEN1 Gly111Val missense mutation in a Chinese pedigree with early-onset Alzheimer’s disease. Neurobiol. Aging.

[43] Pembroke W.G., Hartl C.L., Geschwind D.H. (2021). Evolutionary conservation and divergence of the human brain transcriptome. Genome Biol..

[44] Takami K., Terai K., Matsuo A., Walker D.G., McGeer P.L. (1997). Expression of presenilin-1 and -2 mRNAs in rat and Alzheimer’s disease brains. Brain Res..

[45] Lee M., Slunt H.H., Martin L.J., Thinakaran G., Kim G., Gandy S.E., Seeger M., Koo E., Price D.L., Sisodia S.S. (1996). Expression of Presenilin 1 and 2 (PS1 and PS2) in Human and Murine Tissues. J. Neurosci..

[46] Belloy M.E., Napolioni V., Greicius M.D. (2019). A Quarter Century of APOE and Alzheimer’s Disease: Progress to Date and the Path Forward. Neuron.

[47] Zhao N., Liu C.C., Qiao W., Bu G. (2018). Apolipoprotein E, receptors, and modulation of Alzheimer’s disease. Biol. Psychiatry.

[48] Chen Y., Strickland M.R., Soranno A., Holtzman D.M. (2021). Share Apolipoprotein E: Structural insights and links to Alzheimer disease pathogenesis. Neuron.

[49] Yamazaki Y., Zhao N., Caulfield T.R., Liu C.-C., Bu G. (2019). Apolipoprotein E and Alzheimer disease: Pathobiology and targeting strategies. Nat. Rev. Neurol..

[50] Huebbe P., Rimbach G. (2017). Evolution of human apolipoprotein E (APOE) isoforms: Gene structure, protein function and interaction with dietary factors. Aging Res. Rev..

[51] Yamazaki Y., Liu C.-C., Yamazaki A., Shue F., Martens Y.A., Chen Y., Qiao W., Kurti A., Oue H., Ren Y. (2020). Vascular ApoE4 Impairs Behavior by Modulating Gliovascular Function. Neuron.

[52] Lindahl-Jacobsen R., Tan Q., Mengel-From J., Christensen K., Nebel A., Christiansen L. (2013). Effects of the APOE epsilon2 allele on mortality and cognitive function in the oldest old. J. Gerontol. A Biol. Sci. Med. Sci..

[53] Wang C., Xiong M., Gratuze M., Bao X., Shi Y., Andhey P.S., Manis M., Schroeder C., Yin Z., Madore C. (2021). Selective removal of astrocytic APOE4 strongly protects against tau-mediated neurodegeneration and decreases synaptic phagocytosis by microglia. Neuron.

[54] Michaelson D.M. (2014). APOE ε4: The most prevalent yet understudied risk factor for Alzheimer’s disease. Alzheimers Dement.

[55] Holtzman D.M., Herz J., Bu G. (2012). Apolipoprotein E and Apolipoprotein E Receptors: Normal Biology and Roles in Alzheimer Disease. Cold Spring Harb. Perspect. Med..

[56] Martínez-Morillo E., Hansson O., Atagi Y., Bu G., Minthon L., Diamandis E.P., Nielsen H.M. (2014). Total apolipo-protein E levels and specific isoform composition in cerebrospinal fluid and plasma from Alzheimer’s disease patients and controls. Acta Neuropathol..

[57] Getz G.S., Reardon C.A. (2018). Apoprotein E and Reverse Cholesterol Transport. Int. J. Mol. Sci..

[58] Chapman J., Korczyn A.D., Karussis D.M., Michaelson D.M. (2001). The effects of APOE genotype on age at onset and progression of neurodegenerative diseases. Neurology.

[59] Safieh M., Korczyn A.D., Michaelson D.M. (2019). ApoE4: An emerging therapeutic target for Alzheimer’s disease. BMC Med..

[60] Gao Y., Tan L., Yu J.T., Tan L. (2018). Tau in Alzheimer’s disease: Mechanisms and therapeutic strategies. Curr. Alzheimer Res..

[61] Drummond E., Pires G., MacMurray C., Askenazi M., Nayak S., Bourdon M., Safar J., Ueberheide B., Wisniewski T. (2020). Phosphorylated tau interactome in the human Alzheimer’s disease brain. Brain.

[62] Chong F.P., Ng K.Y., Koh R.Y., Chye S.M. (2018). Tau Proteins and Tauopathies in Alzheimer’s Disease. Cell. Mol. Neurobiol..

[63] Horie K., Barthélemy N.R., Sato C., Bateman R.J. (2020). CSF tau microtubule binding region identifies tau tangle and clinical stages of Alzheimer’s disease. Brain.

[64] Vossel K.A., Xu J.C., Fomenko V., Miyamoto T., Suberbielle E., Knox J.A., Ho K., Kim D.H., Yu G.Q., Mucke L. (2015). Tau reduction prevents Abeta-induced axonal transport deficits by blocking activation of GSK3beta. J. Cell. Biol..

[65] Vossel K.A., Zhang K., Brodbeck J., Daub A.C., Sharma P., Finkbeiner S., Cui B., Mucke L. (2010). Tau reduction prevents Abeta-induced defects in axonal transport. Science.

[66] Elie A., Prezel E., Guérin C., Denarier E., Ramirez-Rios S., Serre L., Andrieux A., Fourest-Lieuvin A., Blanchoin L., Arnal I. (2015). Tau co-organizes dynamic microtubule and actin networks. Sci. Rep..

[67] Prezel E., Elie A., Delaroche J., Stoppin-Mellet V., Bosc C., Serre L., Fourest-Lieuvin A., Andrieux A., Vantard M., Arnal I. (2018). Tau can switch microtubule network organizations: From random networks to dynamic and stable bundles. Mol. Biol. Cell.

[68] Chang C.W., Shao E., Mucke L. (2021). Tau: Enabler of diverse brain disorders and target of rapidly evolving thera-peutic strategies. Science.

[69] Sohn P.D., Huang C.T.-L., Yan R., Fan L., Tracy T.E., Camargo C.M., Montgomery K.M., Arhar T., Mok S.-A., Freilich R. (2019). Pathogenic Tau Impairs Axon Initial Segment Plasticity and Excitability Homeostasis. Neuron.

[70] Milà-Alomà M., Salvadó G., Gispert J.D., Vilor-Tejedor N., Grau-Rivera O., Sala-Vila A., Sánchez-Benavides G., Arenaza-Urquijo E.M., Crous-Bou M., González-de-Echávarri J.M. (2020). Amyloid beta, tau, synaptic, neurodegeneration, and glial biomarkers in the preclinical stage of the Alzheimer’s continuum. Alzheimers Dement.

[71] Iqbal K., Liu F., Gong C.-X. (2015). Tau and neurodegenerative disease: The story so far. Nat. Rev. Neurol..

[72] Sánchez M.P., García-Cabrero A.M., Sánchez-Elexpuru G., Burgos D.F., Serratosa J.M. (2018). Tau-induced pathology in epilepsy and dementia: Notions from patients and animal models. Int. J. Mol. Sci..

[73] Palmqvist S., Tideman P., Cullen N., Zetterberg H.L., Blennow K., Dage J.L., Stomrud E., Janelidze S., Mattsson-Carlgren N., Alzheimer’s Disease Neuroimaging Initiative (2021). Prediction of future Alzheimer’s disease dementia using plasma phosphotau combined with other accessible measures. Nat. Med..

[74] Guo T., Korman D., Baker S.L., Landau S.M., Jagust W.J. (2020). Longitudinal Cognitive and Biomarker Measurements Support a Unidirectional Pathway in Alzheimer’s Disease Pathophysiology. Biol. Psychiatry.

[75] Cheng Y., Bai F. (2018). The association of tau with mitochondrial dysfunction in Alzheimer’s disease. Front Neurosci..

[76] Guha S., Johnson G.V.W., Nehrke K. (2020). The crosstalk between pathological tau phosphorylation and mitochon-drial dysfunction as a key to understanding and treating Alzheimer’s disease. Mol. Neurobiol..

[77] Cisternas P., Taylor X., Lasagna-Reeves C.A. (2019). The amyloid-tau-neuroinflammation axis in the context of cerebral amyloid angiopathy. Int. J. Mol. Sci..

[78] Bryant A.G., Manhard M.K., Salat D.H., Rosen B.R., Hyman B.T., Johnson K.A., Huang S., Bennett R.E., Yen Y.-F. (2021). Heterogeneity of Tau Deposition and Microvascular Involvement in MCI and AD. Curr. Alzheimer Res..

[79] Ashford J.W. (2019). The dichotomy of Alzheimer’s disease pathology: Amyloid-beta and Tau. J. Alzheimers Dis..

[80] Sengoku R. (2020). Aging and Alzheimer’s disease pathology. Neuropathology.

[81] Soldan A., Pettigrew C., Cai Q., Wang J., Wang M.C., Moghekar A., Miller M.I., Albert M., BIOCARD Research Team (2017). Cognitive reserve and long-term change in cognition in aging and preclinical Alzheimer’s disease. Neurobiol Aging.

[82] Mecocci P., Boccardi V., Cecchetti R., Bastiani P., Scamosci M., Ruggiero C., Baroni M. (2018). A long journey into aging, brain aging, and Alzheimer’s disease following the oxidative stress tracks. J. Alzheimers Dis..

[83] Xia X., Jiang Q., McDermott J., Han J.-D.J. (2018). Aging and Alzheimer’s disease: Comparison and associations from molecular to system level. Aging Cell.

[84] Wilkins H.M., Swerdlow R.H. (2021). Mitochondrial links between brain aging and Alzheimer’s disease. Transl. Neurodegener..

[85] Bhatt S., Puli L., Patil C.R. (2020). Role of reactive oxygen species in the progression of Alzheimer’s disease. Drug Discov. Today.

[86] Wang X., Wang W., Li L., Perry G., Lee H.G., Zhu X. (2014). Oxidative stress and mitochondrial dysfunction in Alzheimer’s disease. Biochim. Biophys. Acta.

[87] Moulton M.J., Barish S., Ralhan I., Chang J., Goodman L.D., Harland J.G., Marcogliese P.C., Johansson J.O., Ioannou M.S., Bellen H.J. (2021). Neuronal ROS-induced glial lipid droplet formation is altered by loss of Alzheimer’s dis-ease-associated genes. Proc. Natl. Acad. Sci. USA.

[88] Nesi G., Sestito S., Digiacomo M., Rapposelli S. (2017). Oxidative Stress, mitochondrial abnormalities and proteins deposition: Multitarget approaches in Alzheimer’s disease. Curr. Top Med. Chem..

[89] Luque-Contreras D., Carvajal K., Toral-Rios D., Franco-Bocanegra D., Campos-Peña V. (2014). Oxidative stress and metabolic syndrome: Cause or consequence of Alzheimer’s disease?. Oxid. Med. Cell Longev..

[90] Dansokho C., Heneka M.T. (2017). Neuroinflammatory responses in Alzheimer’s disease. J. Neural Transm..

[91] Minter M.R., Taylor J.M., Crack P.J. (2016). The contribution of neuroinflammation to amyloid toxicity in Alzheimer’s disease. J. Neurochem..

[92] Uddin M.S., Kabir M.T., Jalouli M., Rahman M.A., Jeandet P., Behl T., Alexiou A., Albadrani G.M., Ab-del-Daim M.M., Perveen A. (2022). Neuroinflammatory signaling in the pathogenesis of Alzheimer’s disease. Curr. Neuropharmacol..

[93] Bagyinszky E., Youn Y.C., An S.S.A., Kim S.Y. (2014). The genetics of Alzheimer’s disease. Clin. Interv. Aging.

[94] Leng F., Edison P. (2021). Neuroinflammation and microglial activation in Alzheimer disease: Where do we go from here?. Nat. Rev. Neurol..

[95] Arranz A.M., De Strooper B. (2019). The role of astroglia in Alzheimer’s disease: Pathophysiology and clinical implications. Lancet Neurol..

[96] Rangaraju S., Dammer E.B., Raza S.A., Rathakrishnan P., Xiao H., Gao T., Duong D.M., Pennington M.W., Lah J.J., Seyfried N.T. (2018). Identification and therapeutic modulation of a pro-inflammatory subset of disease-associated-microglia in Alzheimer’s disease. Mol. Neurodegener..

[97] Hagemeyer N., Hanft K.-M., Akriditou M.-A., Unger N., Park E.S., Stanley E.R., Staszewski O., Dimou L., Prinz M. (2017). Microglia contribute to normal myelinogenesis and to oligodendrocyte progenitor maintenance during adulthood. Acta Neuropathol..

[98] Cserép C., Pósfai B., Dénes Á. (2021). Shaping neuronal fate: Functional heterogeneity of direct microglia-neuron interactions. Neuron.

[99] Wang W.Y., Tan M.S., Yu J.T., Tan L. (2015). Role of pro-inflammatory cytokines released from microglia in Alzheimer’s disease. Ann. Transl. Med..

[100] Liddelow S.A., Guttenplan K.A., Clarke L.E., Bennett F.C., Bohlen C.J., Schirmer L., Bennett M.L., Münch A.E., Chung W.-S., Peterson T.C. (2017). Neurotoxic reactive astrocytes are induced by activated microglia. Nature.

[101] Ren S., Breuillaud L., Yao W., Yin T., Norris K.A., Zehntner S.P., D’Adamio L. (2021). TNF-alpha-mediated reduc-tion in in-hibitory neurotransmission precedes sporadic Alzheimer’s disease pathology in young Trem2(R47H) rats. J. Biol. Chem..

[102] Carter S.F., Herholz K., Rosa-Neto P., Pellerin L., Nordberg A., Zimmer E.R. (2019). Astrocyte Biomarkers in Alzheimer’s Disease. Trends Mol. Med..

[103] Fakhoury M. (2018). Microglia and astrocytes in Alzheimer’s disease: Implications for therapy. Curr. Neuropharmacol..

[104] Zeng L., Zhang D., Liu Q., Zhang J., Mu K., Gao X., Zhang K., Li H., Wang Q., Zheng Y. (2021). Alpha-asarone Improves Cognitive Function of APP/PS1 Mice and Reducing Abeta_42_, P-tau and neuroinflammation, and promoting neuron survival in the hippocampus. Neuroscience.

[105] Aguilar-Pineda J.A., Vera-Lopez K.J., Shrivastava P., Chávez-Fumagalli M.A., Nieto-Montesinos R., Alvarez-Fernandez K.L., Mamani L.D.G., Del-Carpio G.D., Gomez-Valdez B., Miller C.L. (2021). Vascular smooth muscle cell dysfunction contribute to neuroinflammation and Tau hyperphosphorylation in Alzheimer disease. iScience.

[106] Oliveira J.D., Kucharska E., Garcez M.L., Rodrigues M.S., Quevedo J., Moreno-Gonzalez I., Budni J. (2021). Inflammatory cascade in Alzheimer’s disease pathogenesis: A review of experimental findings. Cells.

[107] Cantley L.C. (2002). The phosphoinositide 3-kinase pathway. Science.

[108] Frey R.S., Gao X., Javaid K., Siddiqui S.S., Rahman A., Malik A.B. (2006). Phosphatidylinositol 3-kinase gamma signaling through protein kinase Czeta induces NADPH oxidase-mediated oxidant generation and NF-kappaB activation in endothelial cells. J. Biol. Chem..

[109] Zhao L., Vogt P.K. (2008). Class I PI3K in oncogenic cellular transformation. Oncogene.

[110] Yoshioka K. (2021). Class II phosphatidylinositol 3-kinase isoforms in vesicular trafficking. Biochem. Soc. Trans..

[111] Fyffe C., Buus R., Falasca M. (2013). Genetic and epigenetic regulation of phosphoinositide 3-kinase isoforms. Curr. Pharm. Des..

[112] Iacono A., Pompa A., De Marchis F., Panfili E., Greco F.A., Coletti A., Orabona C., Volpi C., Belladonna M.L., Mon-danelli G. (2020). Class IA PI3Ks regulate subcellular and functional dynamics of IDO1. EMBO Rep..

[113] Geering B., Cutillas P.R., Vanhaesebroeck B. (2007). Regulation of class IA PI3Ks: Is there a role for monomeric PI3K subunits?. Biochem. Soc. Trans..

[114] Zhao W., Qiu Y., Kong D. (2016). Class I phosphatidylinositol 3-kinase inhibitors for cancer therapy. Acta Pharm. Sin. B.

[115] Zhu H., Xu Y., Li M., Chen Z. (2021). Inhibition Sequence of miR-205 Hinders the Cell Proliferation and Migration of Lung Cancer Cells by Regulating PETN-Mediated PI3K/AKT Signal Pathway. Mol. Biotechnol..

[116] Chu N., Viennet T., Bae H., Salguero A., Boeszoermenyi A., Arthanari H., Cole P.A. (2020). The structural determinants of PH domain-mediated regulation of Akt revealed by segmental labeling. elife.

[117] Guo J.P., Coppola D., Cheng J.Q. (2016). IKBKE protein activates Akt independent of phosphatidylinositol 3-kinase/PDK1/mTORC2 and the pleckstrin homology domain to sustain malignant transformation. J. Biol. Chem..

[118] Thapa N., Chen M., Horn H.T., Choi S., Wen T., Anderson R.A. (2020). Phosphatidylinositol-3-OH kinase signalling is spatially organized at endosomal compartments by microtubule-associated protein 4. Nat. Cell Biol..

[119] Pearce L.R., Komander D., Alessi D.R. (2010). The nuts and bolts of AGC protein kinases. Nat. Rev. Mol. Cell Biol..

[120] Gagliardi P.A., Puliafito A., Primo L. (2017). PDK1: At the crossroad of cancer signaling pathways. Semin. Cancer Biol..

[121] Mora A., Komander D., van Aalten D., Alessi D.R. (2004). PDK1, the master regulator of AGC kinase signal transduction. Semin. Cell Dev. Biol..

[122] Hossen M.J., Kim S.C., Yang S., Kim H.G., Jeong D., Yi Y.-S., Sung N.Y., Lee J.-O., Kim J.-H., Cho J.Y. (2015). PDK1 disruptors and modulators: A patent review. Expert Opin. Ther. Patents.

[123] Raimondi C., Falasca M. (2011). Targeting PDK1 in cancer. Curr. Med. Chem..

[124] Bayascas J.R. (2008). Dissecting the role of the 3-phosphoinositide-dependent protein kinase-1 (PDK1) signalling pathways. Cell Cycle.

[125] Komander D., Kular G., Deak M., Alessi D.R., van Aalten D.M. (2005). Role of T-loop phosphorylation in PDK1 activation, stability, and substrate binding. J. Biol. Chem..

[126] Wick M.J., Ramos F.J., Chen H., Quon M., Dong L.Q., Liu F. (2003). Mouse 3-Phosphoinositide-dependent Protein Kinase-1 Undergoes Dimerization and trans-Phosphorylation in the Activation Loop. J. Biol. Chem..

[127] Gómez-Suárez M., Gutiérrez-Martínez I.Z., Hernández-Trejo J.A., Hernández-Ruiz M., Suárez-Pérez D., Candelario A., Kamekura R., Medina-Contreras O., Schnoor M., Ortiz-Navarrete V. (2016). 14-3-3 Proteins regulate Akt Thr308 phosphorylation in intestinal epithelial cells. Cell Death Differ..

[128] Dan H.C., Antonia R., Baldwin A.S. (2016). PI3K/Akt promotes feedforward mTORC2 activation through IKKα. Oncotarget.

[129] Busschots K., Lopez-Garcia L.A., Lammi C., Stroba A., Zeuzem S., Piiper A., Alzari P.M., Neimanis S., Arencibia J.M., Engel M. (2012). Substrate-Selective Inhibition of Protein Kinase PDK1 by Small Compounds that Bind to the PIF-Pocket Allosteric Docking Site. Chem. Biol..

[130] Gao X., Harris T.K. (2006). Steady-state Kinetic Mechanism of PDK1. J. Biol. Chem..

[131] Balendran A., Casamayor A., Deak M., Paterson A., Gaffney P., Currie R., Downes C., Alessi D.R. (1999). PDK1 acquires PDK2 activity in the presence of a synthetic peptide derived from the carboxyl terminus of PRK2. Curr. Biol..

[132] Jafari M., Ghadami E., Dadkhah T., Akhavan-Niaki H. (2018). PI3k/AKT signaling pathway: Erythropoiesis and beyond. J. Cell. Physiol..

[133] Cecconi S., Mauro A., Cellini V., Patacchiola F. (2012). The role of Akt signalling in the mammalian ovary. Int. J. Dev. Biol..

[134] Degan S.E., Gelman I.H. (2021). Emerging Roles for AKT Isoform Preference in Cancer Progression Pathways. Mol. Cancer Res..

[135] Calleja V., Laguerre M., Larijani B. (2012). Role of the C-terminal regulatory domain in the allosteric inhibition of PKB/Akt. Adv. Biol. Regul..

[136] Song G., Ouyang G., Bao S. (2005). The activation of Akt/PKB signaling pathway and cell survival. J. Cell. Mol. Med..

[137] Alessi D.R., Andjelkovic M., Caudwell B., Cron P., Morrice N., Cohen P., Hemmings B.A. (1996). Mechanism of activation of protein kinase B by insulin and IGF-1. EMBO J..

[138] Garza J.C., Guo M., Zhang W., Lu X.-Y. (2012). Leptin restores adult hippocampal neurogenesis in a chronic unpre-dictable stress model of depression and reverses glucocorticoid-induced inhibition of GSK-3β/β-catenin signaling. Mol. Psychiatry.

[139] Wang D., Chen J., Chen H., Duan Z., Xu Q., Wei M., Wang L., Zhong M. (2011). Leptin regulates proliferation and apoptosis of colorectal carcinoma through PI3K/Akt/mTOR signalling pathway. J. Biosci..

[140] Cowan C.M., Bossing T., Page A., Shepherd D., Mudher A. (2010). Soluble hyperphosphorylated tau causes micro-tubule breakdown and functionally compromises normal tau in vivo. Acta Neuropathol..

[141] Lippens G., Sillen A., Landrieu I., Amniai L., Sibille N., Barbier P., Leroy A., Hanoulle X., Wieruszeski J.M. (2007). Tau aggregation in Alzheimer’s disease: What role for phosphorylation?. Prion.

[142] Yan Y., Huang H. (2019). Interplay Among PI3K/AKT, PTEN/FOXO and AR Signaling in Prostate Cancer. Adv. Exp. Med. Biol..

[143] Farhan M., Wang H., Gaur U., Little P., Xu J., Zheng W. (2017). FOXO Signaling Pathways as Therapeutic Targets in Cancer. Int. J. Biol. Sci..

[144] Kitagishi Y., Nakanishi A., Ogura Y., Matsuda S. (2014). Dietary regulation of PI3K/AKT/GSK-3β pathway in Alzheimer’s disease. Alzheimer’s Res. Ther..

[145] Jaworski J., Spangler S., Seeburg D.P., Hoogenraad C., Sheng M. (2005). Control of Dendritic Arborization by the Phosphoinositide-3′-Kinase-Akt-Mammalian Target of Rapamycin Pathway. J. Neurosci..

[146] Kumar V., Zhang M.-X., Swank M.W., Kunz J., Wu G.-Y. (2005). Regulation of Dendritic Morphogenesis by Ras-PI3K-Akt-mTOR and Ras-MAPK Signaling Pathways. J. Neurosci..

[147] Wang Q., Liu L., Pei L., Ju W., Ahmadian G., Lu J., Wang Y., Liu F., Wang Y.T. (2003). Control of Synaptic Strength, a Novel Function of Akt. Neuron.

[148] Horwood J.M., Dufour F., Laroche S., Davis S. (2006). Signalling mechanisms mediated by the phosphoinositide 3-kinase/Akt cascade in synaptic plasticity and memory in the rat. Eur. J. Neurosci..

[149] Griffin R.J., Moloney A., Kelliher M., Johnston J.A., Ravid R., Dockery P., O’Connor R., O’Neill C. (2005). Activa-tion of Akt/AKT, increased phosphorylation of Akt substrates and loss and altered distribution of Akt and PTEN are features of Alzheimer’s disease pathology. J. Neurochem..

[150] Moloney A.M., Griffin R.J., Timmons S., O’Connor R., Ravid R., O’Neill C. (2010). Defects in IGF-1 receptor, insulin receptor and IRS-1/2 in Alzheimer’s disease indicate possible resistance to IGF-1 and insulin signaling. Neurobiol. Aging.

[151] Sonoda Y., Mukai H., Matsuo K., Takahashi M., Ono Y., Maeda K., Akiyama H., Kawamata T. (2010). Accumula-tion of tu-mor-suppressor PTEN in Alzheimer neurofibrillary tangles. Neurosci. Lett..

[152] Cassidy L., Fernandez F., Johnson J.B., Naiker M., Owoola A.G., Broszczak D.A. (2020). Oxidative stress in alzheimer’s disease: A review on emergent natural polyphenolic therapeutics. Complement Ther. Med..

[153] O’Neill C., Kiely A.P., Coakley M.F., Manning S., Long-Smith C.M. (2012). Insulin and IGF-1 signaling: Longevity, protein homoeostasis and Alzheimer’s disease. Biochem. Soc. Trans..

[154] Talbot K., Wang H.Y., Kazi H., Han L.Y., Bakshi K.P., Stucky A., Fuino R.L., Kawaguchi K.R., Samoyedny A.J., Wilson R.S. (2012). Demonstrated brain insulin resistance in Alzheimer’s disease patients is associated with IGF-1 resistance, IRS-1 dysregulation, and cognitive decline. J. Clin Invest..

[155] Liu F., Grundke-Iqbal I., Iqbal K., Gong C.-X. (2005). Contributions of protein phosphatases PP1, PP2A, PP2B and PP5 to the regulation of tau phosphorylation. Eur. J. Neurosci..

[156] Neill C.O. (2013). PI3-kinase/Akt/mTOR signaling: Impaired on/off switches in aging, cognitive decline and Alzheimer’s disease. Exp. Gerontol..

[157] Willette A.A., Johnson S.C., Birdsill A.C., Sager M.A., Christian B., Baker L.D., Craft S., Oh J., Statz E., Hermann B.P. (2015). Insulin resistance predicts brain amyloid deposition in late middle-aged adults. Alzheimer’s Dement.

[158] Razani E., Pourbagheri-Sigaroodi A., Safaroghli-Azar A., Zoghi A., Shanaki-Bavarsad M., Bashash D. (2021). The PI3K/Akt signaling axis in Alzheimer’s disease: A valuable target to stimulate or suppress?. Cell Stress Chaperones..

[159] Gao X., Harris T.K. (2006). Role of the PH domain in regulating in vitro autophosphorylation events required for reconstitution of PDK1 catalytic activity. Bioorg. Chem..

[160] Sancak Y., Thoreen C.C., Peterson T.R., Lindquist R.A., Kang S.A., Spooner E., Carr S.A., Sabatini D.M. (2007). PRAS40 Is an Insulin-Regulated Inhibitor of the mTORC1 Protein Kinase. Mol. Cell.

[161] Chen Y., Li Y., Hsieh T., Wang C., Cheng K., Wang L., Lin T., Cheung C.H.A., Wu C., Chiang H. (2019). Aging-induced Akt activation involves in aging-related pathologies and Aβ-induced toxicity. Aging Cell.

[162] Zhang Y.-Y., Mei Z., Wu J.-W., Wang Z.-X. (2008). Enzymatic Activity and Substrate Specificity of Mitogen-activated Protein Kinase p38α in Different Phosphorylation States. J. Biol. Chem..

[163] Chang K.H., de Pablo Y., Lee H.P., Lee H.G., Smith M.A., Shah K. (2010). Cdk5 is a major regulator of p38 cascade: Relevance to neurotoxicity in Alzheimer’s disease. J. Neurochem..

[164] Chen B., Teng Y., Zhang X., Lv X., Yin Y. (2016). Metformin Alleviated Aβ-Induced Apoptosis via the Suppression of JNK MAPK Signaling Pathway in Cultured Hippocampal Neurons. BioMed Res. Int..

[165] Lee J.K., Kim N.J. (2017). Recent advances in the inhibition of p38 MAPK as a potential strategy for the treatment of Alzheimer’s disease. Molecules.

[166] Wang H.Q., Sun X.B., Xu Y.X., Zhao H., Zhu Q.Y., Zhu C.Q. (2010). Astaxanthin upregulates heme oxygenase-1 expression through ERK1/2 pathway and its protective effect against β-amyloid-induced cytotoxicity in SH-SY5Y cells. Brain Res..

[167] Zhao L., Wang J.L., Wang Y.R., Fa X.Z. (2013). Apigenin attenuates copper-mediated β-amyloid neurotoxicity through antioxidation, mitochondrion protection and mapk signal inactivation in an ad cell model. Brain Res..

[168] Dresselhaus E.C., Boersma M.C.H., Meffert M.K. (2018). Targeting of NF-κB to dendritic spines is required for synaptic signaling and spine development. J. Neurosci..

[169] Yu Z., Zhou D., Bruce-Keller A.J., Kindy M.S., Mattson M.P. (1999). Lack of the p50 subunit of nuclear factor-kappaB increases the vulnerability of hippocampal neurons to excitotoxic injury. J. Neurosci..

[170] Chen C.H., Zhou W., Liu S., Deng Y., Cai F., Tone M., Tone Y., Tong Y., Song W. (2012). Increased NF-kappaB signalling up-regulates BACE1 expression and its therapeutic potential in Alzheimer’s disease. Int. J. Neuropsychopharmacol..

[171] Shi Z., Hong Y., Zhang K., Wang J., Zheng L., Zhang Z., Hu Z., Han X., Han Y., Chen T. (2017). BAG-1M coactivates BACE1 transcription through NF-kappaB and accelerates Abeta production and memory deficit in Alzheimer’s disease mouse model. Biochim. Biophys. Acta Mol. Basis. Dis..

[172] Cheng F., Fransson L., Mani K. (2020). Proinflammatory cytokines induce accumulation of glypican-1-derived heparan sulfate and the C-terminal fragment of β-cleaved APP in autophagosomes of dividing neuronal cells. Glycobiology.

[173] Yu H.-J., Koh S.-H. (2017). The role of PI3K/AKT pathway and its therapeutic possibility in Alzheimer’s disease. Hanyang Med. Rev..

[174] Oomura Y., Hori N., Shiraishi T., Fukunaga K., Takeda H., Tsuji M., Matsumiya T., Ishibashi M., Aou S., Li X. (2006). Leptin facilitates learning and memory performance and enhances hippocampal CA1 long-term potentiation and CaMK II phosphorylation in rats. Peptides.

[175] Harvey J., Solovyova N., Irving A. (2006). Leptin and its role in hippocampal synaptic plasticity. Prog. Lipid Res..

[176] Duvall A., Gallicchio V. (2017). Lithium treatment in clinical medicine: History, current status and future use. J. Cell. Sci. Ther..

[177] Komander D., Fairservice A., Deak M., Kular G.S., Prescott A., Downes C.P., Safrany S., Alessi D., van Aalten D. (2004). Structural insights into the regulation of PDK1 by phosphoinositides and inositol phosphates. EMBO J..

[178] Bayascas J.R., Wullschleger S., Sakamoto K., García-Martínez J.M., Clacher C., Komander D., van Aalten D.M.F., Boini K.M., Lang F., Lipina C. (2008). Mutation of the PDK1 PH Domain Inhibits Protein Kinase B/Akt, Leading to Small Size and Insulin Resistance. Mol. Cell. Biol..

[179] Zurashvili T., Cordon-Barris L., Ruiz-Babot G., Zhou X., Gomez N., Gimenez-Llort L., Bayascas J.R. (2013). Inter-action of PDK1 with phosphoinositides is essential for neuronal differentiation but dispensable for neuronal survival. Mol. Cell Biol..

[180] Najafov A., Shpiro N., Alessi D.R. (2012). Akt is efficiently activated by PIF-pocket- and PtdIns(3,4,5)P3-dependent mechanisms leading to resistance to PDK1 inhibitors. Biochem. J..

[181] Zhou X., Cordon-Barris L., Zurashvili T., Bayascas J.R. (2014). Fine-tuning the intensity of the AKT/Akt signal enables diverse physiological responses. Cell Cycle.

[182] Giménez-Llort L., Santana-Santana M., Bayascas J.R. (2020). The impact of the PI3K/Akt signaling pathway in anxi-ety and working memory in young and middle-aged PDK1 K465E knock-in mice. Front Behav. Neurosci..

[183] Akhondzadeh S., Noroozian M., Mohammadi M., Ohadinia S., Jamshidi A.H., Khani M. (2003). Salvia officinalis extract in the treatment of patients with mild to moderate Alzheimer’s disease: A double blind, randomized and placebo-controlled trial. J. Clin. Pharm. Ther..

[184] Wang C., Zhang X., Teng Z., Zhang T., Li Y. (2014). Downregulation of PI3K/Akt/mTOR signaling pathway in curcumin-induced autophagy in APP/PS1 double transgenic mice. Eur. J. Pharmacol..

[185] Xiong N., Jia M., Chen C., Xiong J., Zhang Z., Huang J., Hou L., Yang H., Cao X., Liang Z. (2011). Potential autophagy enhancers attenuate rotenone-induced toxicity in SH-SY5Y. Neuroscience.

[186] Zhang L., Fang Y., Cheng X., Lian Y.J., Xu H.L., Zeng Z.S., Zhu H.C. (2017). TRPML1 participates in the progression of Alzheimer’s disease by regulating the PPARγ/AMPK/Mtor signalling pathway. Cell Physiol. Biochem..

[187] Tramutola A., Lanzillotta C., Di Domenico F. (2016). Targeting mTOR to reduce Alzheimer-related cognitive decline: From current hits to future therapies. Expert Rev. Neurother..

[188] Zeng Y., Zhang J., Zhu Y.G., Zhang J., Shen H., Lu J.P., Pan X.D., Lin N., Dai X.M., Zhou M. (2015). Tripchlorolide improves cognitive deficits by reducing amyloid β and upregulating synapse-related proteins in a transgenic model of Alzheimer’s Disease. J. Neurochem..

[189] Shen Y.T., Zhang Q., Gao X.R., Ding F. (2011). An active fraction of Achyranthes bidentata polypeptides prevents apoptosis in-duced by serum deprivation in SH-SY5Y cells through activation of PI3K/AKT/Gsk3β pathways. Neurochem. Res..

[190] Zaytseva Y.Y., Valentino J.D., Gulhati P., Evers B.M. (2012). mTOR inhibitors in cancer therapy. Cancer Lett..

[191] Ahmed A.R., Candeo A., D’Abrantes S., Needham S.R., Yadav R.B., Botchway S.W., Parker A.W. (2020). Directly imaging the localisation and photosensitization properties of the panmTOR inhibitor, AZD2014, in living cancer cells. J. Photochem. Photobiol. B.

[192] Manterola L., Hernando-Rodríguez M., Ruiz A., Apraiz A., Arrizabalaga O., Vellón L., Alberdi E., Cavaliere F., Lacerda H.M., Jimenez S. (2013). 1-42 β-amyloid peptide requires PDK1/nPKC/Rac 1 pathway to induce neuronal death. Transl. Psychiatry.

[193] Santana-Santana M., Bayascas J.-R., Giménez-Llort L. (2021). Fine-Tuning the PI3K/Akt Signaling Pathway Intensity by Sex and Genotype-Load: Sex-Dependent Homozygotic Threshold for Somatic Growth but Feminization of Anxious Phenotype in Middle-Aged PDK1 K465E Knock-In and Heterozygous Mice. Biomedicines.

[194] Gooz M. (2010). ADAM-17: The enzyme that does it all. Crit. Rev. Biochem. Mol. Biol..

[195] Edwards D.R., Handsley M.M., Pennington C.J. (2008). The ADAM metalloproteinases. Mol. Asp. Med..

[196] Ezpeleta J., Baudouin V., Arellano-Anaya Z.E., Boudet-Devaud F., Pietri M., Baudry A., Haeberlé A.-M., Bailly Y., Kellermann O., Launay J.-M. (2019). Production of seedable Amyloid-β peptides in model of prion diseases upon PrP Sc-induced PDK1 overactivation. Nat. Commun..

[197] Palumbo S., Paterson C., Yang F., Hood V.L., Law A.J. (2021). PKBbeta/AKT2 deficiency impacts brain mTOR sig-naling, pre-frontal cortical physiology, hippocampal plasticity and select murine behaviors. Mol. Psychiatry.

